# LysoTracker and MitoTracker Red are transport substrates of P‐glycoprotein: implications for anticancer drug design evading multidrug resistance

**DOI:** 10.1111/jcmm.13485

**Published:** 2018-01-26

**Authors:** Benny Zhitomirsky, Hodaya Farber, Yehuda G. Assaraf

**Affiliations:** ^1^ Department of Biology The Fred Wyszkowski Cancer Research Laboratory Technion‐Israel Institute of Technology Haifa Israel

**Keywords:** P‐glycoprotein, LysoTracker, MitoTracker, cancer, drug‐resistance

## Abstract

LysoTracker and MitoTracker Red are fluorescent probes widely used for viable cell staining of lysosomes and mitochondria, respectively. They are utilized to study organelle localization and their resident proteins, assess organelle functionality and quantification of organelle numbers. The ATP‐driven efflux transporter P‐glycoprotein (P‐gp) is expressed in normal and malignant tissues and extrudes structurally distinct endogenous and exogenous cytotoxic compounds. Thus, once aromatic hydrophobic compounds such as the above‐mentioned fluorescent probes are recognized as transport substrates, efflux pumps including P‐gp may abolish their ability to reach their cellular target organelles. Herein, we show that LysoTracker and MitoTracker Red are expelled from P‐gp‐overexpressing cancer cells, thus hindering their ability to fluorescently mark target organelles. We further demonstrate that tariquidar, a potent P‐gp transport inhibitor, restores LysoTracker and MitoTracker Red cell entry. We conclude that LysoTracker and MitoTracker Red are P‐gp transport substrates, and therefore, P‐gp expression must be taken into consideration prior to cellular applications using these probes. Importantly, as MitoTracker was a superior P‐gp substrate than LysoTracker Red, we discuss the implications for the future design of chemotherapeutics evading cancer multidrug resistance. Furthermore, restoration of MitoTracker Red fluorescence in P‐gp‐overexpressing cells may facilitate the identification of potent P‐gp transport inhibitors (*i.e*. chemosensitizers).

## Introduction

Fluorescence microscopy plays a major role in the field of cell biology and physiology. A wide array of viable molecular markers that target specific organelles and emit fluorescence are an integral part of the toolbox of modern biomedical research. However, various fluorescent dyes were previously shown to be expelled from cells *via* ATP‐driven efflux pumps, thus preventing them from reaching their target organelles and limiting their application 1, 2, 3.

**Figure 1:**
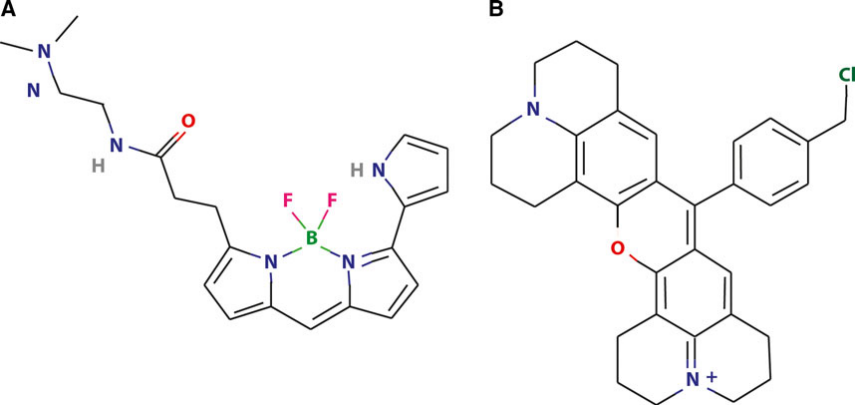
The molecular structures of LysoTracker Red (**A**) and MitoTracker Red (**B**). Adapted from pubchem.ncbi.nlm.nih.gov.

LysoTracker Red DND‐99 is a hydrophobic weak base and a viable fluorescent marker, which selectively accumulates in acidic vesicular compartments, predominantly in late endosomes and lysosomes 4. LysoTracker Red has a wide spectrum of applications in the field of cell biology and fluorescence microscopy, and is often used to detect and mark lysosomes in viable cells. This probe has an excitation wavelength of 576 nm and emission of 590 nm. LysoTracker Red contains several aromatic rings with a positively charged nitrogen at acidic pH (Fig. 1A). Due to its hydrophobic nature (Log P = 2.1 5), LysoTracker Red enters cells *via* simple diffusion. Remarkably, owing to the acidic pH of the lysosome, upon entry into the lysosome, LysoTracker Red becomes protonated and hence is markedly sequestrated in lysosomes. Applications of LysoTracker Red include, for example, colocalization of fluorescent drugs or fluorescently marked proteins with lysosomes to demonstrate their lysosomal localization 6, 7, tracking the localization of lysosomes within the cell 8 and quantification of the number of lysosomes in cells using fluorescence microscopy and computational analysis 9. Moreover, due to the fact that lysosomal acidity plays a crucial role in the accumulation of LysoTracker inside this acidic organelle, its fluorescence intensity may serve as an indication of alterations in lysosomal pH 10. Furthermore, a recent study suggested that LysoTracker dyes can be used for flow cytometric studies of autophagy, demonstrating that LysoTracker Green signal is enhanced during autophagic process in a manner comparable to the *bona fide* autophagy marker LC3B in Jurkat T‐cell leukaemia and K562 erythromyeloid leukaemia cells 11, 12.

MitoTracker Red CMXRos is a red fluorescent dye which accumulates in mitochondria in viable cells 13 and has an excitation wavelength of 578 nm and emission of 599 nm. The hydrophobic (Log P = 1.15 14) cationic properties of MitoTracker Red allow it to selectively accumulate in mitochondria due to the negative mitochondrial membrane potential. MitoTracker Red has a large multi‐aromatic structure with two positively charged nitrogens (Fig. 1B). MitoTracker Red has a wide spectrum of applications including the study of the morphology and functional state of mitochondria 15, quantification of the number of mitochondria per cell 16, colocalization of proteins within the organelle 17 and detection of apoptotic cells using flow cytometric analysis *via* decreased fluorescence of MitoTracker Red due to mitochondrial disintegration 18.

The ATP‐binding cassette (ABC) is a superfamily of ATP‐driven efflux transporters, containing multiple transmembrane proteins that actively translocate multiple substrates across biological membranes 19, 20. ABC transporters play an important physiological role in protecting cells from numerous endogenous and exogenous toxic compounds and as such have been found to contribute to cancer multidrug resistance (MDR) by preventing various chemotherapeutic agents from entering cancer cells and exerting their cytotoxic activity 18, 21. The first member of the ABC superfamily to be identified was P‐glycoprotein (P‐gp) 22, 23, 24, the product of the MDR1 gene. P‐gp displays a poly‐specific substrate recognition pattern hence extruding a wide spectrum of structurally and mechanistically distinct molecules in a wide range of molecular weights ranging from 330 to 4000 Da 25, 26, 27. The majority of P‐gp substrates are hydrophobic and thus markedly accumulate in the plasma membrane. It is well‐established that P‐gp acts as a hydrophobic ‘vacuum cleaner’ pump which binds hydrophobic substrates dissolved in the plasma membrane hence extrude them out of the cell, thus abolishing their entry into the cytoplasm and their arrival at their cellular target sites 28, 29, 30. P‐gp has two transmembrane domains containing 12 transmembrane helices, which serve as the substrate binding sites 25. P‐gp forms a natural barrier against toxins and xenobiotics in various human organs, but slight alterations such as polymorphisms in exons of the MDR1 gene may endow this pump with the ability to recognize different anticancer drugs 31, thus, rendering tumour cells MDR to chemotherapy 32, 33. In addition to various cytotoxins and chemotherapeutic agents, ABC transporters including P‐gp were also shown to expel various molecular markers, such as the viable DNA dye Hoechst 33342, the oxidative stress indicator CellROX, the mitochondrial marker MitoTracker Green and the mitochondrial membrane potential probe JC‐1 2, 34. This extrusion of fluorescent molecular markers hinders their application in cells expressing these MDR pumps resulting in misinterpretation of the findings if this extrusion is not taken into account.

Here, we employed human P‐gp‐overexpressing cancer cell lines to demonstrate that the two widely used fluorescent probes LysoTracker Red and MitoTracker Red are transport substrates of P‐gp, hence hindering their ability to fluorescently mark lysosomes and mitochondria in P‐gp‐overexpressing cells, respectively. We further demonstrate that these molecular stains are not substrates of two other MDR pumps—breast cancer resistance protein (BCRP) and multidrug resistance protein‐1 (MRP1).

## Materials and methods

### Chemicals

LysoTracker Red DND99 and MitoTracker Red CMXRos were purchased from Invitrogen (Carlsbad, CA, USA). Hoechst 33342 was obtained from Molecular Probes (Eugene, OR, USA). Tariquidar was purchased from MedKoo Bioscience (Chapel Hill, NC, USA). KO143 was purchased from Sigma‐Aldrich (St. Louis, MO, USA), and MK571 was purchased from Cayman Chemicals (Ann Arbor, MI, USA).

### Cell culture

The cell lines used in this study were as follows: SW‐1573 human lung carcinoma cells and their doxorubicin‐resistant subline SW‐1573/2R160 35; EPG85‐257P human gastric carcinoma cells and their daunorubicin‐resistant subline EPG85‐257‐RDB 36; 2008 human ovarian carcinoma cells and their subline 2008/MRP1 stably infected to overexpress the ABCC1 gene 37; A549 non‐small cell lung cancer cells and their MDR BCRP‐overexpressing subline A549/K1.5 38. All cell lines used in this study were maintained in RPMI‐1640 medium (Gibco, Paisley, UK), supplemented with 10% foetal bovine serum, 2 mM glutamine, 100 μg/ml penicillin and streptomycin (Biological Industries, Beit HaEmek, Israel) in a humid atmosphere containing 5% CO_2_ at 37°C.

### Membrane protein extraction and Western blot analysis

Membrane proteins were extracted from the different tumour cell lines as previously described 39. Protein content was determined using the Bio‐Rad protein assay. P‐gp, MRP1 and BCRP protein levels, as normalized to Na^+^/K^+^‐ATPase, were determined by Western blot using JSB‐1 anti‐P‐gp, MRPr1 anti‐MRP1 and BXP‐53 anti BCRP monoclonal antibodies (kindly provided by Prof. R. J. Scheper and Dr. G. L. Scheffer, VU University Medical Center, Amsterdam, The Netherlands) and the affinity purified polyclonal anti‐Na^+^/K^+^‐ATPase antibody (KETTY, kindly provided by Prof. S. J. D. Karlish) as previously described 39. The membrane was then reacted with horseradish‐peroxidase conjugated secondary antibodies (Jackson Immunoresearch Labs, West Grove, PA, USA) and enhanced chemiluminescence (ECL) detection was performed according to the manufacturer's instructions (Biological Industries).

### Live cell microscopy

For live cell imaging, tumour cells were plated in 24‐well glass bottom plates (*In Vitro* Scientific, CA, USA) at a concentration of 10^5^ cells/well in 1 ml growth medium and allowed to attach overnight. Cells were incubated for 1 hr with 100 nM LysoTracker Red or 100 nM MitoTracker Red at 37°C, in the presence or absence of the appropriate ABC transporter inhibitors; 100 nM Tariquidar for P‐gp‐expressing cells, 20 μM MK571 for MRP1‐expressing cells and 700 nM Ko143 for BCRP‐expressing cells. To achieve nuclear DNA staining prior to fluorescence imaging, cells were incubated with 2 μg/ml Hoechst 33,342 in growth medium for 10 min. Subcellular fluorescence was detected using the InCell analyser 2000 (GE Healthcare Bio‐Sciences, Pittsburgh, PA, USA) fluorescence microscope.

### Computational and statistical analysis

Computational analysis of LysoTracker Red and MitoTracker Red fluorescence intensity was determined using the InCell investigator software, analysing 20 fields from each well, containing at least 200 cells. The statistical analysis of computational results was performed with one‐tailed‐coupled Student's *t*‐test. *P*‐value lower than 0.05 was considered statistically significant.

## Results

**Figure 2:**
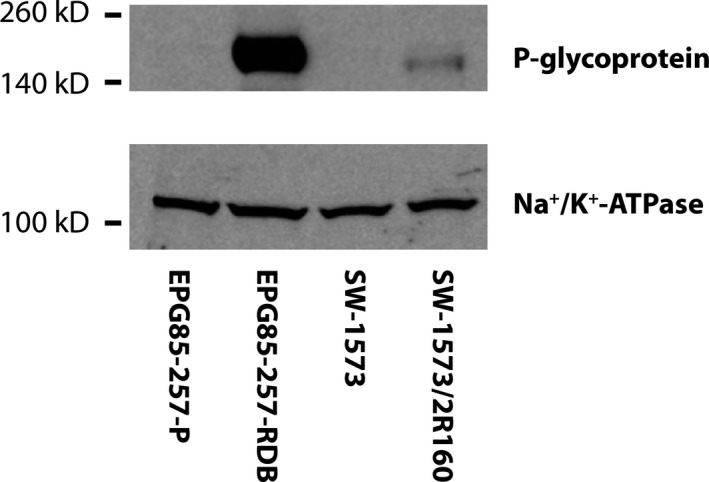
**The human multidrug‐resistant cancer cell lines EPG85‐257‐RDB and SW‐1573/2R160 express P‐glycoprotein.** Membrane proteins were extracted from parental EPG85‐257P cells and their MDR subline EPG85‐257‐RDB, as well as parental SW‐1573 cells and their cognate MDR derivative SW‐1573/2R160 cells. P‐gp expression was determined by Western blot analysis using the P‐gp antibody JSB‐1. Anti‐Na^+^/K^+^‐ATPase was used to evaluate the actual protein loading.

To determine whether or not LysoTracker Red and MitoTracker Red are transport substrates of P‐gp, SW‐1573 human lung carcinoma cells and their doxorubicin‐resistant P‐gp‐expressing subline SW‐1573/2R160 35, as well as EPG85‐257P human gastric carcinoma cells and their daunorubicin‐resistant P‐gp‐overexpressing subline EPG85‐257‐RDB 36 were used. P‐gp levels in these paired tumour cell lines were determined by Western blot analysis (Fig. 2). Neither of the parental tumour cell lines expressed any detectable levels of P‐gp, whereas SW‐1573/2R160 cells displayed a relatively low expression level of P‐gp which appeared as a ~170 kD protein; in contrast, EPG85‐257‐RDB expressed very high levels of P‐gp (Fig. 2).

**Figure 3:**
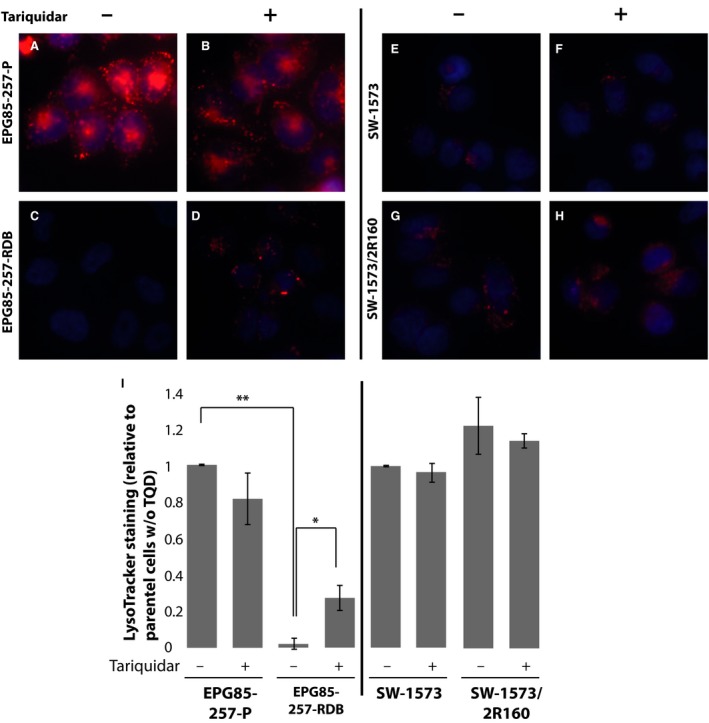
**LysoTracker Red is extruded from cells *via* P‐gp.** EPG85‐257P, EPG85‐257‐RDB, SW‐1573 and SW‐1573/2R160 cells were incubated with LysoTracker Red (100 nM) for 1 hr, in the presence or absence of tariquidar (100 nM). Hoechst 33342 (2 μg/ml) was used to stain nuclei. Cellular fluorescence was followed by an InCell Analyzer fluorescence microscope (**A**–**H**). LysoTracker Red fluorescence intensity was quantified using InCell investigator software. Error bars indicate standard deviation (**I**). *Student's *t*‐test *P*‐value < 0.05 **P‐value < 0.01.

To determine whether or not LysoTracker and MitoTracker Red are transport substrates of P‐gp, these two pairs of cognate tumour cell lines were incubated with 100 nM of each of the two fluorescent probes for 1 hr in the presence or absence of the potent P‐gp transport inhibitor tariquidar (100 nM) 40. Lysosomes in parental EPG85‐257P cells were successfully stained with LysoTracker Red (Fig. 3, Panels A and I) and tariquidar had no significant effect on lysosome staining in these P‐gp‐lacking cells (Fig. 3, Panels B and I). In contrast, LysoTracker Red failed to stain lysosomes in P‐gp‐overexpressing EPG85‐257‐RDB cells, and no detectable red fluorescence was found in these cells (Fig. 3 Panels C and I). Co‐incubation of EPG85‐257‐RDB cells with LysoTracker Red and tariquidar partially restored the lysosomal LysoTracker staining (Fig. 3 Panels D and I) when compared to the fluorescence intensity in parental EPG85‐257P cells. These results indicate that LysoTracker Red is a substrate of P‐gp, and hence, cells expressing high levels of this efflux pump will not be stained by LysoTracker without inhibition of P‐gp efflux activity. The partial restoration of LysoTracker staining in EPG85‐257‐RDB cells incubated with tariquidar might be due to partial inhibition of P‐gp or due to actual lower lysosome content in these cells compared to their parental cell line. Surprisingly, no such effect was found in P‐gp‐expressing SW‐1573/2R160 cells, as the level of LysoTracker fluorescence in these cells was similar to the level of LysoTracker staining in parental SW‐1573 cells and was not significantly altered by addition of tariquidar (Fig. 3 Panels E‐H, and Panel I). This result suggests that the moderate P‐gp levels in SW‐1573/2R160 cells were not sufficient to efficiently extrude LysoTracker Red from these MDR cells.

**Figure 4:**
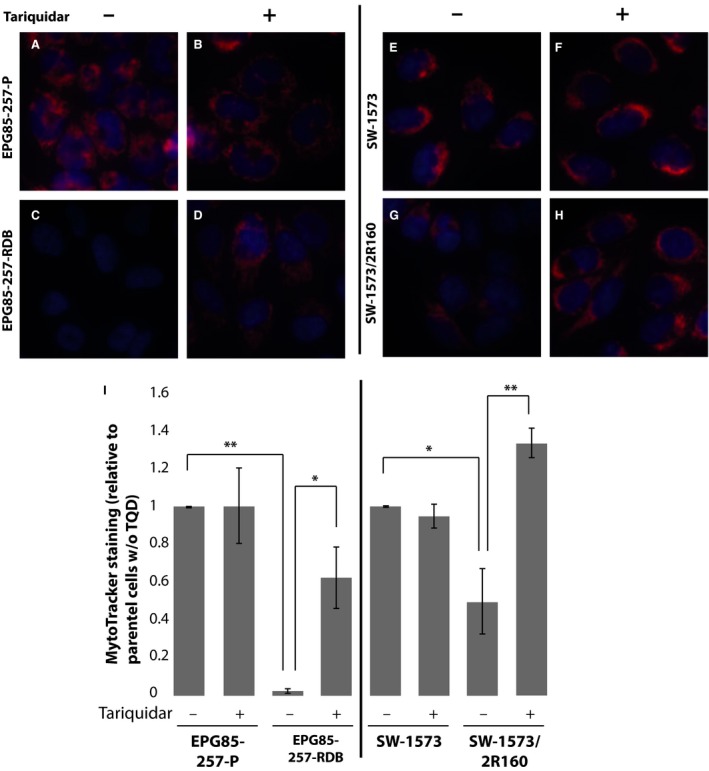
**MitoTracker Red is secreted from cells *via* P‐gp.** EPG85‐257P, EPG85‐257‐RDB, SW‐1573 and SW‐1573/2R160 cells were incubated with MitoTracker Red (100 nM) for 1 hr, in the presence or absence of tariquidar (100 nM). Hoechst 33342 (2 μg/ml) was used to stain nuclei. Cellular fluorescence was studied using an InCell Analyzer fluorescence microscope (**A**–**H**). MitoTracker Red fluorescence intensity was quantified using InCell investigator software. Error bars indicate standard deviation (**I**). *Student's *t*‐test *P*‐value < 0.05 **P‐value < 0.01.

MitoTracker Red successfully stained mitochondria in parental EPG85‐257P cells (Fig. 4, Panels A and I), and this fluorescence staining was not affected by tariquidar (Fig. 4, Panels B and I). In contrast, MitoTracker Red failed to stain P‐gp‐overexpressing EPG85‐257‐RDB cells in which no MitoTracker fluorescence was detected (Fig. 4, Panels C and I). Similar to LysoTracker Red staining, MitoTracker Red staining in EPG85‐257‐RDB cells was partially restored in cells co‐incubated with tariquidar, indicating that MitoTracker Red is also a transport substrate of P‐gp (Fig. 4 Panels D and I). The partial restoration of MitoTracker Red staining by tariquidar in EPG85‐257‐RDB cells, combined with the partial restoration of LysoTracker Red staining, suggests that tariquidar only partially inhibited P‐gp transport activity in these cells. Unlike with LysoTracker Red, MitoTracker Red staining was significantly reduced in SW‐1573/2R160 cells when compared to parental SW‐1573 cells and was successfully restored by co‐incubation with tariquidar (Fig. 4, Panels E‐H and I), suggesting that MitoTracker Red is a much better P‐gp substrate than LysoTracker Red, as even the low levels of P‐gp in SW‐1573/2R160 cells were sufficient to significantly reduce MitoTracker accumulation in these cells.

**Figure 5:**
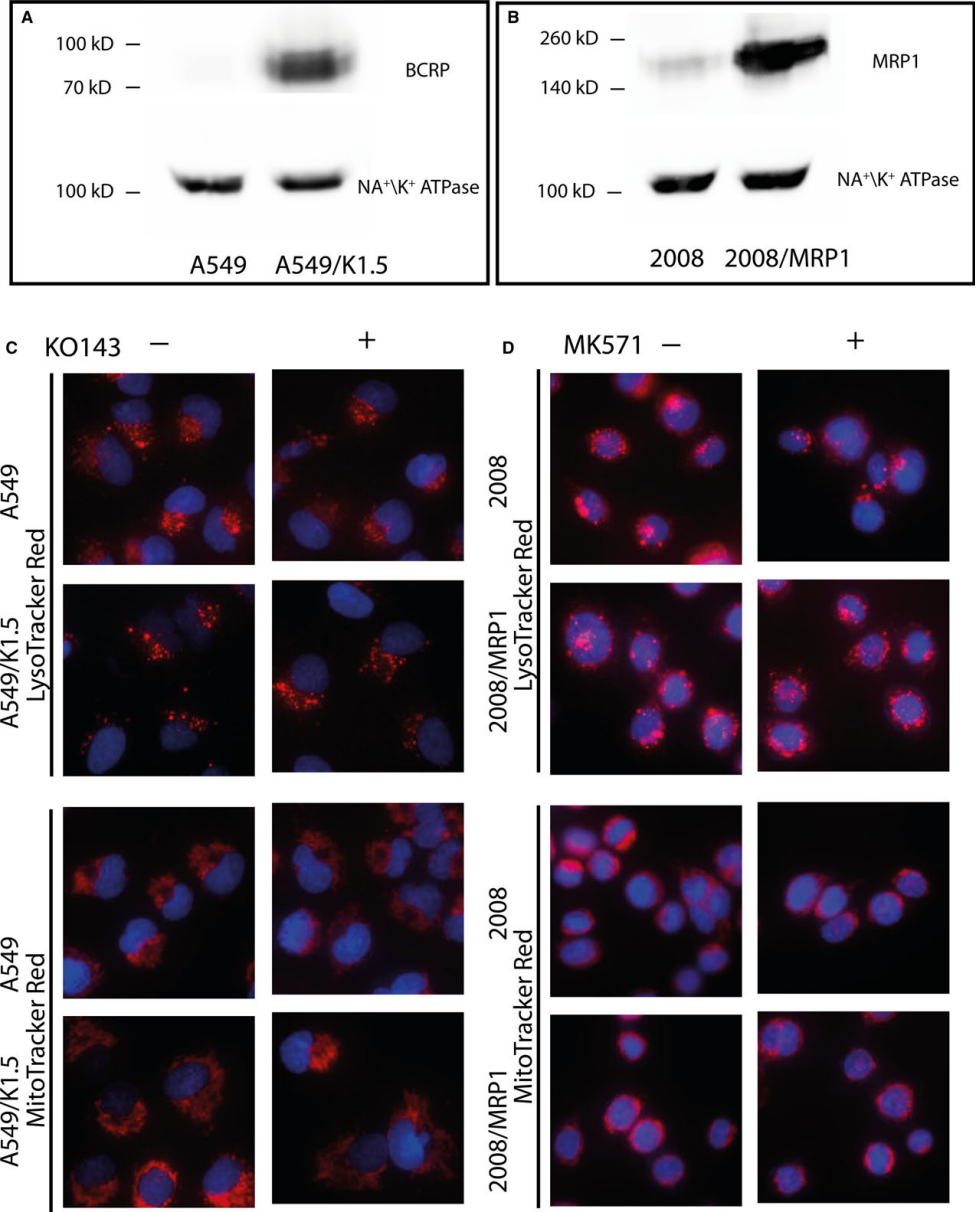
**LysoTracker Red and MitoTracker Red are neither substrates of MRP1 nor of BCRP.** BCRP levels in A549 and A549/K1.5 cells (**A**) and MRP1 levels in 2008 and 2008/MRP1 cells (**B**) were determined using Western blot analysis. A549 and A549/K1.5 cells were incubated with LysoTracker Red or MitoTracker Red (100 nM) for 1 hr, with or without co‐incubation with Ko143 (0.7 μM) (**C**). 2008 and 2008/MRP1 cells were incubated with LysoTracker Red or MitoTracker Red (100 nM) for 1 hr, with or without co‐incubation with MK571 (20 μM) (**D**). Hoechst 33342 (2 μg/ml) was used to stain nuclei. Cellular fluorescence was determined using an InCell Analyzer fluorescence microscope.

**Figure 6:**
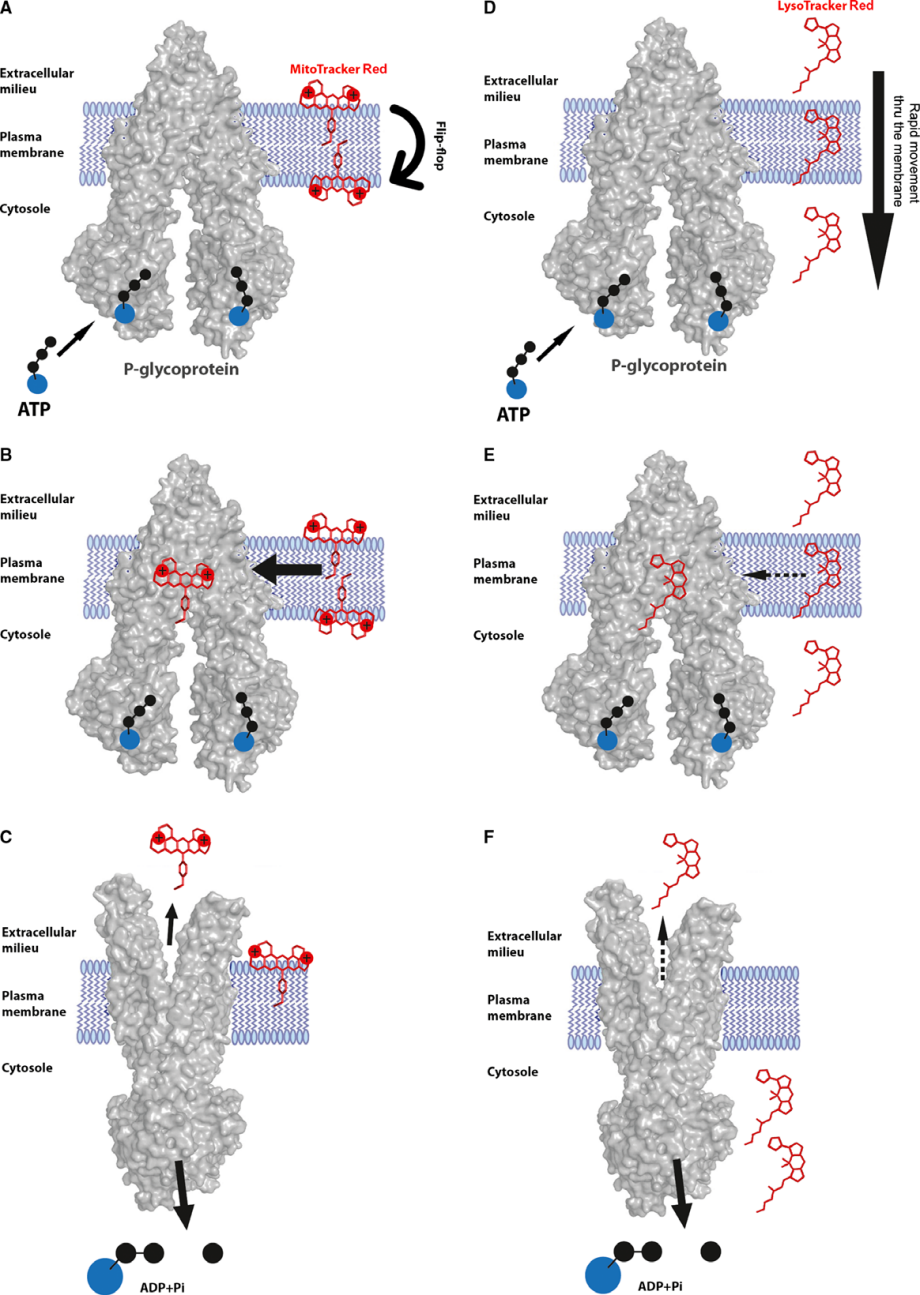
**A proposed model for P‐gp‐mediated MitoTracker Red and LysoTracker Red extrusion from the plasma membrane.** MitoTracker Red is initially incorporated into the outer leaflet of the plasma membrane. Due to the positive charges on the nitrogens, MitoTracker Red is presumably able to translocate into the inner cytoplasmic leaflet *via* a relatively slow flip‐flop mechanism (**A**). This prolongs the residence time of MitoTracker Red within the plasma membrane, allowing P‐gp to efficiently bind it (**B**) and efficiently extrude it from the cell (**C**). LysoTracker Red directly and rapidly traverses the plasma membrane, resulting in a relatively short period of retention within the lipid bilayer (**D**). The fast transport of LysoTracker Red across the membrane markedly reduces the ability of P‐gp to bind it (**E**), resulting in a less efficient extrusion from the cell (**F**).

We have further determined whether or not LysoTracker Red and MitoTracker Red are substrates of other major efflux pumps of the ABC superfamily including MRP1 and BCRP. To this end, we employed 2008/MRP1 human ovarian carcinoma cells stably infected to overexpress the ABCC1 gene which encodes for the MDR efflux transporter MRP1 37 and the MDR non‐small cell lung cancer cells A549/K1.5 which overexpress the MDR pump BCRP encoded by the ABCG2 gene 38. The protein levels of MRP1 and BCRP were determined in these cell lines using Western blot analysis (Fig. 5 Panels A, B); this confirmed that 2008/MRP1 and A549/K1.5 cells overexpressed MRP1 and BCRP, respectively. Fluorescence staining experiments with LysoTracker Red and MitoTracker Red were performed as described above. MK571 (20 μM) was used as an MRP1 transport inhibitor in 2008/MRP1 cells, whereas Ko143 (700 nM) was used as a BCRP transport inhibitor in A459/K1.5 cells (Fig. 6). Both LysoTracker Red and MitoTracker Red successfully stained lysosomes and mitochondria, respectively, in both parental and MDR tumour cell lines. Inhibition of MRP1 in 2008/MRP1 cells as well as inhibition of BCRP in A459/K1.5 cells did not alter LysoTracker and MitoTracker Red staining. These results demonstrate that these fluorescent probes are neither substrates of MRP1 nor of BCRP.

## Discussion

Our present findings establish that the viable intracellular fluorescent markers LysoTracker Red and MitoTracker Red are transport substrates of P‐gp. From a physiological perspective, P‐gp is expressed at substantial levels in different tissues such as the liver, brain, kidney, pancreas, colon and adrenal gland where it is actively involved in the ATP‐dependent extrusion of toxic metabolites and anticancer drugs into the bile, urine and the lumen of the gastrointestinal tract as well as the blood 41. P‐gp plays a central role in maintaining epithelial and endothelial cell barrier function and therefore is overexpressed in the blood–brain barrier, blood‐CSF barrier, blood‐testis barrier as well as the maternal‐foetal barrier in the placenta 42, 43, 44. P‐gp was also found in haematopoietic stem cells and lymphocytes, suggesting a role in protecting them from endogenous and exogenous toxicants 45, 46. P‐gp is also overexpressed in various tumour cells, mostly in colon, renal, breast, ovarian and adrenal carcinomas 19, 46, 47. Furthermore, selection with structurally and mechanistically distinct cytotoxic drugs has been previously shown to provoke P‐gp overexpression due to either stable gene amplification or transcriptional activation, rendering these malignant cells resistant to multiple chemotherapeutics. For example, treatment with the following cytotoxic drugs: vinblastine 48, doxorubicin 49, mefloquine 50 and colchicine triggered P‐gp overexpression in tumour cells 51. In contrast, some compounds have been shown to reduce P‐gp expression including curcumin 52, achieving the opposite result, suppressing MDR of tumour cells to drugs which are P‐gp transport substrates.

Expulsion of LysoTracker and MitoTracker Red from P‐gp‐expressing cells leads to markedly reduced accumulation of these fluorescent markers in their target organelles, hence significantly decreasing organelle fluorescence. The ability to fluorescently label and evaluate quantitative and qualitative features of lysosomes or mitochondria in P‐gp‐overexpressing cells would be seriously limited or completely abolished. In that case, markedly reduced fluorescence will not be a true indication of the cellular lysosome or mitochondria content and/or function, but rather a result of the viable fluorescent markers being extruded from the cells *via* P‐gp. Thus, the staining of P‐gp‐expressing cells with these molecular markers may lead to erroneous interpretation of results. The use of LysoTracker dyes in flow cytometry for the evaluation of autophagy that was recently suggested 11, 12 might also lead to misinterpretation of results if fluorescent dyes that are P‐gp substrates are used for this aim in P‐gp‐expressing cells. In such experiments, P‐gp‐expressing cells will appear to display lower levels of autophagy when in fact the reduced staining is due to P‐gp‐mediated extrusion of the LysoTracker dye. Such misinterpretation of results might lead, for example, to false conclusions regarding the mechanism underlying anticancer resistance in various cancer cells. Combining these facts with the broad expression of P‐gp in various tissues and MDR cancer cell lines and tumour cell lines suggests that P‐gp‐expression must be seriously taken into consideration when staining cells with LysoTracker and MitoTracker Red, if quantification and functionality of organelle fluorescence is of importance for the interpretation of the data.

While P‐gp is predominantly located on the plasma membrane, several studies have also found P‐gp to also be localized in the membranes of intracellular organelles including lysosomes 53, 54, 55. A recent study revealed that P‐gp may be localized in the lysosomal membrane and contribute to MDR by actively sequestering drugs into lysosomes, thus preventing them from reaching their cellular target 56. In the context of the current study, lysosomal membrane‐targeted P‐gp is expected to achieve the opposite effect of plasma membrane P‐gp by actively transporting LysoTracker into lysosomes, as opposed to the dye's primary mechanism of accumulation *via* simple diffusion and lysosomal accumulation through cation trapping. The latter possibility will enhance LysoTracker fluorescence, hence creating an additional pathway for the misinterpretation of quantitative data. Thus, not only the expression level of P‐gp has to be taken into consideration when staining cells with LysoTracker Red or MitoTracker Red but also its subcellular localization.

While our results demonstrate that it is possible to overcome P‐gp‐mediated extrusion of LysoTracker or MitoTracker Red from cells using P‐gp transport inhibitors such as tariquidar, the inhibition of P‐gp might be partial resulting in unreliable quantitative and analytical data. We thus suggest testing of the cells under study for P‐gp expression prior to their staining with LysoTracker or MitoTracker Red and choosing different tumour cell lines or different molecular stains if P‐gp expression is detected.

Our findings reveal that MitoTracker Red was a much superior P‐gp transport substrate than LysoTracker Red; this is strongly supported by the fact that human lung carcinoma SW‐1573/2R160 cells with low P‐gp overexpression readily expelled MitoTracker Red but failed to extrude LysoTracker Red. While these two fluorescent organelle probes are polyaromatic hydrophobic compounds, a close examination of their structures raises plausible biophysical explanations for this remarkable difference between MitoTracker Red as a *bona fide* P‐gp transport substrate and LysoTracker Red being a relatively poor P‐gp substrate. MitoTracker Red is a hydrophobic compound (Log P = 1.15) 14, 56 containing a large planar quinolizino quinoline ring structure in which two nitrogens are positively charged at physiological pH (Fig. 1), where the chloromethylphenyl group is also contained within this planar structure. In this respect, we have previously shown that such large planar hydrophobic aromatic structures, including anticancer drugs with positive charge(s), traverse the plasma membrane relatively slowly *via* a flip‐flop mechanism 56, 57, 58, 59, 60, 61. This slow flip‐flip involves an initial insertion of the drug in the outer leaflet of the plasma membrane with the positively charged residues protruding to the extracellular milieu; this is followed by a relatively slow flip‐flop into the cytoplasmic membrane leaflet (Fig. 6A). Previous studies have shown that the residence half‐life times in the outer leaflet of the membrane for various polyaromatic anthrayclines can range from 6 sec. for idarubicin to as long as 6 min. for mitoxantrone. Thus, as P‐gp can readily recognize polyaromatic hydrophobic structures with a slight cationic charge and as P‐gp displays a remarkable transport substrate turnover number of 900 per second 14, 30, 56, it will have ample time to bind MitoTracker Red within the lipid bilayer and efficiently extrude it. In contrast, LysoTracker Red is a much smaller hydrophobic molecule (Log P = 2.1) 5, 10, 56 containing a tri‐pyrrole core as well as a highly flexible aliphatic propanamidato side chain, which harbours a terminal nitrogen that may or may not be positively charged at physiological pH. Consequently, the expected residence time of this relatively small hydrophobic molecule (that is not necessarily charged at physiological pH) in the plasma membrane is expected to be rather short, hence leaving no realistic membrane residence time for substrate binding and extrusion by P‐gp.

These considerations of P‐gp transport substrate recognition, demonstrated herein by the differences between LysoTracker Red and MitoTracker Red structures and the influence of these structural differences on the efficiency of P‐gp‐mediated extrusion of these fluorescent dyes from P‐gp‐expressing cells, bear important implications for the future design of anticancer drugs. From a pharmacological perspective, when designing novel small molecule hydrophobic anticancer drugs, one should avoid the introduction of positively charged residues and/or other moieties that may enhance plasma membrane residence time, hence leaving for P‐gp ample time to recognize and efficiently extrude such novel chemotherapeutics, resulting in cancer MDR. Consistently, previous studies have shown that acceleration of passive drug transbilayer movement across the plasma membrane by local anaesthetics markedly abolished the ability of P‐gp to recognize and extrude *bona fide* P‐gp substrates, thus overcoming MDR 56, 62. Another important possible implication of the current study is that these fluorescent probes could be used in a bioassay for high‐throughput drug screening protocols that would facilitate the development of potent P‐gp transport inhibitors known as MDR chemosensitizers. Thus, large chemical libraries could be readily screened in P‐gp‐overexpressing cells while searching for efficient restoration of MitoTracker Red fluorescence, which would be used as an indication for successful inhibition of P‐gp efflux activity, thus facilitating the development of MDR chemosensitizers for effective reversal of P‐gp‐mediated cancer MDR, which continues to be a major hindrance to curative chemotherapy of various human malignancies.

## Conflict of interests

The authors confirm that there is no conflict of interests.
